# The mediating effect of self-efficacy on family functioning and psychological resilience in prostate cancer patients

**DOI:** 10.3389/fpsyg.2024.1392167

**Published:** 2024-05-20

**Authors:** Yao Zhou, Honglan Shan, Cuigan Wu, Haiyan Chen, Yuanyuan Shen, Wenying Shi, Lina Wang, Qinghe Li

**Affiliations:** ^1^Department of Urology, The Yancheng School of Clinical Medicine of Nanjing Medical University, Yancheng, China; ^2^The Third People's Hospital of Yancheng, The Affiliated Hospital of Jiangsu Vocational College of Medicine, Yancheng, China; ^3^Nursing Department, The Yancheng School of Clinical Medicine of Nanjing Medical University and The Third People's Hospital of Yancheng, Yancheng, China; ^4^Department of Oncology, The Yancheng School of Clinical Medicine of Nanjing Medical University, Yancheng, China; ^5^Department of Neurosurgery, The Yancheng School of Clinical Medicine of Nanjing Medical University, Yancheng, China

**Keywords:** self-efficacy, family functioning, psychological resilience, mediation, prostate cancer

## Abstract

**Aims:**

Prostate cancer patients face impaired body image and psychological distress during the diagnosis and treatment of the disease, which leads to changes in mood, cognition and behavior. Psychological resilience has been shown to buffer shocks and stresses from the disease. Therefore, this study investigates the relationship between family functioning and psychological resilience in prostate cancer patients and the mediating role of self-efficacy between family functioning and psychological resilience to provide a relevant theoretical basis for improving patients' psychological status by providing relevant theoretical basis.

**Method:**

Using a cross-sectional design, participants were 215 patients with prostate cancer admitted to and treated in a tertiary hospital in Jiangsu province, China. Questionnaires were administered using the general information questionnaire, the Connor-Davidson Resilience Scale (CD-RISC), the Family Adaptation, Partnership, Growth, Affection, and Resolution Index (APGAR), and the General Self-efficacy Scale (GSES). Data were analyzed using descriptive and correlational analyses and the bootstrap mediation test was used to test the effect relationship between the variables.

**Results:**

Family functioning, self-efficacy and psychological resilience were significantly and positively correlated (*r* = 0.526, *P* < 0.01; *r* = 0.378, *P* < 0.01; *r* = 0.358, *P* < 0.01). The mediating effect of psychological resilience between family functioning and psychological resilience was significant, accounting for 42.56%.

**Conclusion:**

Family function and self-efficacy have been shown to increase the level of psychological resilience in prostate cancer patients. Attention should be paid to the mental health problems of prostate cancer patients, early screening and intervention, and the use of patients' family resources to improve their confidence in recovering from the disease, thus increasing their psychological resilience and improving their mental health.

## 1 Introduction

Prostate cancer is a malignancy that is more common in the older male population. The current ranking of malignancy incidence in the male population shows that prostate cancer ranks second (Sung et al., 2021), making it a potential threat to men's health. The survival rate of patients is approximately 70% from the time of diagnosis to 5 years post treatment (Clegg et al., 2002). However, a better prognosis for the disease does not mean that this group does not need attention, and this group still faces multiple physical and psychological burdens.

In addition to the cancer itself, patients may face a variety of complications such as metastasis, cancer pain, lower urinary tract symptoms, and impaired body image from androgen deprivation therapy (ADT) (Chambers et al., 2017), which includes not only changes in physical appearance, but also avoiding others, diminished sensory attractiveness, and altered sense of self, diminished sensory attractiveness (Hopwood et al., 2001; Bowie et al., 2022), and altered sense of self (Sebri et al., 2021), all of which can be distressing to the patient, resulting in emotional, cognitive, and behavioral changes (Brookman-May et al., 2019; Culp et al., 2020). From a physiological perspective, previous studies have found that disease stress affects the molecular mechanisms of the apoptotic pathway in prostate cancer cells themselves, leading to type-specific proliferation of prostate cancer cells (Hassan et al., 2013), while negatively impacting men with prostate cancer's health outcomes (Chrobak et al., 2023). Therefore, understanding the disease burden and psychological distress faced by prostate cancer patients in advance may help to target intervention strategies more accurately (Zhang W. et al., 2023).

Whereas traditional psychology emphasizes “trauma-stress-disadaptation”, meaning that research on stress and coping has historically focused on highlighting the risks associated with negative outcomes (Sturgeon and Zautra, 2013), the concept of positive psychology, which has flourished in modern psychology, emphasizes positive adaptation, shifting attention already to positive traits and strategies that can promote resilience (Richardson, 2002). Psychological resilience is at the heart of positive psychology, and refers to an individual's ability to adapt to their environment and return to their original state in the face of stress or challenges, helping them to reintegrate and restore balance in their thinking in the face of adversity (Richardson, 2002). The resilience factor is a distinct characteristic that promotes wellbeing regardless of risk; when a person is able to demonstrate calm resolution strategies in the face of stress and distress, this implies that they have positive character and personality traits. Resilient individuals have a more flexible thought structure than vulnerable individuals, and they draw support from internal and environmental resources (e.g., family support), which helps them to recover from cancer as quickly as possible and distinguishes them from those who are too affected by the disease to escape it (Mcewen, 2017). Psychological resilience minimizes the negative effects of stress, both physically and psychologically. Research has shown that psychological resilience can help prostate cancer patients better adapt to the changes in body image and social status brought about by the disease (Sharpley et al., 2018; Öcalan and Üzar-Özçetin, 2022), diminish feelings of shame associated with side effects of the disease, and increase male self-esteem and self-confidence (Bowie et al., 2022), buffer depression caused by the stress of diagnosis and treatment (Sharpley et al., 2021), thereby helping the individual to regain calm and optimism.

As the immediate environment in which an individual lives, the family and its support network are an important source of support for the individual. Although the family is considered the smallest unit of society, in traditional Chinese culture the family unit is based on blood ties and family ethics, playing a significant role in a patient's illness and psychological recovery (Wu et al., 2022). The buffering model of social support suggests that good support protects individuals from mitigating stimuli from external stressors and strain (Yildirim et al., 2018). A well-functioning family can provide sufficient material and emotional support to individuals so that patients can receive better treatment options and more attentive care, allowing patients to develop hope in life and pursue meaning in life; secondly, a good family atmosphere can enable families to maintain emotional ties in the face of developmental stress (Nam et al., 2016). Conversely, patients with lower family functioning have a weaker sense of belonging and do not receive positive feedback and emotional support from the outside world, which is not conducive to mitigating the negative effects of adversity (Vogel, 2001; Li et al., 2023). Good family functioning has been shown to improve patients' self-efficacy, help them adopt positive coping strategies, and promote a positive change in their mindset (Xu et al., 2021; Zhang et al., 2021).

Self-efficacy is defined as the degree of self-confidence and affirmation of one's skills and abilities in successfully completing a particular task and meeting one's own expectations (Maddux, 1995). Bandura's theory of self-efficacy suggests that high levels of self-efficacy serve to improve an individual's perception of the illness, increase their ability to adapt to the illness, and thus improve their quality of life. From an assertiveness perspective, self-efficacy is essentially an exploration of an individual's ability to manage and cope with stress, to which psychological resilience itself, as the ability to cope with adversity, is closely related. Patients with a strong sense of self-efficacy can make themselves more confident in the face of stress and in the fight against illness through the dynamic response process of motivation-cognition-choice-emotion (Náfrádi et al., 2017), thus increasing their resilience to the stress of illness (Wang et al., 2023). Self-efficacy can help patients develop positive attitudes and reduce psychological distress (Philip et al., 2013). In cancer patients, several studies have shown a positive association between self-efficacy and psychological resilience in patients with lung, colorectal, and gynecological cancers (Liu et al., 2018; Huang et al., 2019).

Some studies have already examined the relationship between self-efficacy as a mediating variable between social support, negative affect and quality of life in prostate cancer patients (Wang et al., 2022; Martín-Núñez et al., 2023). A study showed that self-efficacy in young lung cancer patients mediated social support and work initiation behaviors, and that young lung cancer survivors with adequate family and social support were able to feel understood and cared for by others, face challenges with optimism, and have the confidence to overcome difficulties. Patients with inadequate perceived support felt vulnerable and incapable of facing emotional and physical challenges (Zhong et al., 2023). Cheng found that self-efficacy in palliative cancer caregivers also mediated the relationship between unmet needs and quality of life (Cheng et al., 2023).

However, the relationship between self-efficacy, family functioning and psychological resilience in prostate cancer patients has not been clarified, and the resilience in illness model (RIM) constructed by Haase et al. (1999) suggests that from a positive psychology perspective, there is a prior association between family protective factors, personal protective factors and psychological resilience, and studies have confirmed that external factors such as family support and self-efficacy can be identified as important components in improving psychological resilience components of psychological resilience. Therefore, this study proposes two hypotheses based on the RIM model:

Hypothesis 1: There is an interrelationship between family functioning, psychological resilience and self-efficacy in prostate cancer patients.

Hypothesis 2: Self-efficacy moderates the relationship between family functioning and psychological resilience in prostate cancer patients.

## 2 Methods

### 2.1 Participants and procedures

In this study, a cross-sectional survey method was used. Prostate cancer patients admitted to a urology ward or attending an outpatient clinic between October 2022 and November 2023 were invited to complete a questionnaire using convenient sampling. The inclusion criteria were as follows. (1) Patients who had been diagnosed with prostate cancer and were receiving treatment. (2) Patients who were receiving or had completed at least one treatment, such as radical prostatectomy, radiotherapy, chemotherapy, endocrine therapy, etc.; (3) Patients who are knowledgeable about prostate cancer treatment. (4) Normal communication and understanding. Exclusion criteria included: (1) suffering from severe mental disorders; (2) having a combination of vital organ damage or other life-threatening diseases. Two hundred and thirty-three questionnaires were distributed and 230 questionnaires were returned, of which 15 questionnaires were incomplete and were excluded from the analyses. Finally, 215 questionnaires were analyzed.

### 2.2 Measures

#### 2.2.1 General information questionnaires

A self-developed general information questionnaire was used to collect relevant information about the patients, including age, level of education, marital status, family residence, financial situation, duration of illness and treatment modality.

#### 2.2.2 Psychological resilience

Psychological resilience was assessed using the Connor-Davidson Resilience Scale (CD-RISC), a 25-item three-dimensional scale consisting of a 13-item toughness dimension (responding calmly to challenges and standing firm), an 8-item strength dimension (returning to normal levels after trials and tribulations or even gaining experiences that help you grow), and a 4-item optimism dimension (dealing positively with difficulties and believing you can overcome them) (Connor and Davidson, 2003). The scale is based on a five-point Likert scale from 0 to 5, with the higher the score, the better the level of psychological resilience. The reliability of this scale in the present study was 0.927.

#### 2.2.3 Family functioning

The Family Adaptation, Partnership, Growth, Affection and Resolution Index (APGAR) (Smilkstein, 1984) is used to assess family functioning and measure individual satisfaction with the family as a whole. The scale has five items and can be divided into five dimensions: Adaptation, Partnership, Growth, Affection and Resolve. The scale is scored on a 3-point scale from 0 (rarely) to 2 (often). A total score of 0–3 means that there is a severe lack of care in the family, a score of 4–6 means that the family is functioning at a moderate level, and a sub-score of 7–10 means that the family is in a good environment with more integration. The reliability of this scale in the present study was 0.813.

#### 2.2.4 Self-esteem

The General Self-Efficacy Scale (GSES) was developed in 1981 by Cheung and Sun (Zhang and Schwarzer, 1995) and has been widely used in Chinese community populations and clinical patients. There are a total of 10 items and scores are rated on a Likert scale from 1 (not at all true) to 4 (completely true). The scores were divided into different levels according to the scores: 19 and below represented a lower level of individuals, the range 20–30 represented a medium level of individuals, and 31 and above represented a higher level. The higher the score, the stronger the sense of belief. The reliability of this scale in this study was 0.87.

### 2.3 Ethical considerations

The principles of voluntariness, confidentiality and harmlessness are strictly adhered to in this study to ensure that the information obtained from the study is used only for scientific research and that complete confidentiality is maintained in relation to the privacy of the subjects. Subjects were given the opportunity to withdraw from the study at any time. The study was approved by the hospital ethics committee during the pre-investigation period under approval number (NO.2022-68).

### 2.4 Data analysis

The data collected were processed and analyzed using IBM SPSS Statistics 26.0. Patients' general and disease-related characteristics were analyzed using descriptive statistics of frequencies and percentages. Self-efficacy, psychological resilience and family function scores were analyzed using means and standard deviations. Pearson correlations were used to analyze correlations between factors. The bootstrap program plug-in of the Process operation macro compiled by Preacher and Hayes (2004) was used to test the mediating effect of self-efficacy in the relationship between family function and psychological resilience. The test level was α = 0.05.

## 3 Results

### 3.1 General demographic data

A total of 215 prostate cancer patients participated in this study, with an age range of 36–90 years, of whom 158 (73.5%) were older adults, and only 27 (12.6%) were under 60 years of age. Seventy-eight (36.3%) lived in towns, 60 (27.9%) in rural areas and 77 (35.8%) in cities. Seventy-three (34%) had a primary school education or less and the majority (81.9%) were married. The vast majority had an average monthly income of 4,000 or more (79.1%). The treatment modality was laparoscopic radical prostatectomy in 167 patients (77.7%), with a smaller proportion treated with open radical prostatectomy (9.8%) or endocrine therapy (12.6%). More than 70% of patients had been diagnosed <3 months previously. Disease stage was concentrated in stage II (43.4%) and stage III (33.5%) (Table 1).

**Table 1:** CD-RISC, APGAR, and GSES scores of prostate cancer patients with different demographic characteristics.

**Variables**	***n* (%)**	**CD-RISC**	**APGAR**	**GSES**
**Age**				
<60	27 (12.6)	69.15 ± 13.76	7.37 ± 2.32	26.33 ± 3.88
60–74	158 (73.5)	62.10 ± 15.47	6.63 ± 2.49	25.20 ± 4.62
≥75	30 (13.9)	55.33 ± 14.39	6.93 ± 2.43	24.47 ± 3.94
*F*		5.928	1.089	2.272
*P*		0.003^**^	0.339	0.282
**Residence**				
Rural	60 (27.9)	57.73 ± 17.75	7.07 ± 2.59	24.45 ± 5.22
Town	78 (36.3)	58.38 ± 12.86	6.39 ± 2.53	24.63 ± 3.97
City	77 (35.8)	70.11 ± 12.59	6.92 ± 2.26	26.39 ± 4.03
*F*		19.106	1.477	4.898
*P*		<0.01^**^	0.231	0.008^**^
**Education**				
Primary or below	73 (34.0)	54.90 ± 12.42	6.40 ± 2.57	23.92 ± 4.20
Middle school	109 (50.7)	63.12 ± 11.16	6.72 ± 2.53	25.43 ± 4.53
College or above	33 (15.3)	74.24 ± 9.15	7.76 ± 1.69	27.58 ± 3.76
*F*		21.855	3.582	8.392
*P*		<0.01^**^	0.03^*^	<0.01^**^
**Marital status**				
Married	176 (81.9)	64.23 ± 15.03	7.028 ± 2.48	25.53 ± 4.34
Unmarried/divorced/widowed	39 (18.1)	52.18 ± 13.64	5.615 ± 2.57	23.94 ± 4.79
*t*		−4.602	−3.312	−2.205
*P*		<0.01^**^	<0.01^**^	0.044^*^
**Monthly family income (**¥**)**				
<4,000	45 (20.9)	56.06 ± 13.96	6.47 ± 2.68	24.11 ± 5.43
4,000–8,000	107 (49.8)	58.12 ± 13.43	6.48 ± 2.40	24.79 ± 3.98
>8,000	63 (29.3)	72.89 ± 11.82	7.47 ± 2.29	26.84 ± 4.06
*F*		27.724	3.724	6.376
*P*		<0.01^**^	0.026^*^	<0.01^**^
**Treatment modality**				
Laparoscopic radical prostatectomy	167 (77.7)	64.17 ± 15.13	6.92 ± 2.40	25.71 ± 4.45
Open radical prostatectomy	21 (9.8)	57.28 ± 13.25	7.01 ± 2.31	24.95 ± 4.51
Endocrine therapy	27 (12.6)	52.59 ± 12.88	5.67 ± 2.82	22.63 ± 3.49
*F*		8.106	3.174	5.847
*P*		<0.01^**^	0.044^*^	<0.01^**^
**Time of disease diagnosis**				
≤ 1 month	53 (24.7)	60.58 ± 16.19	7.56 ± 2.31	25.69 ± 4.03
2–3 months	112 (52.1)	63.41 ± 13.16	6.21 ± 2.34	24.86 ± 4.34
≥3 months	50 (23.2)	65.16 ± 16.06	6.78 ± 2.14	22.74 ± 3.98
*F*		2.076	1.568	5.567
*P*		0.127	0.201	<0.01^**^
I	25 (11.6)	63.94 ± 16.23	7.83 ± 2.79	26.01 ± 4.12
II	93 (43.3)	64.17 ± 16.01	6.75 ± 1.89.	24.98 ± 4.17
III	72 (33.5)	60.57 ± 15.19	6.44 ± 2.03	22.79 ± 3.65
IV	25 (11.6)	59.50 ± 15.74	6.67 ± 2.15	22.53 ± 3.64
*F*		1.784	2.304	6.304
*P*		0.170	0.135	<0.01^**^

^**^P < 0.01, ^*^P < 0.05.

### 3.2 Psychological resilience, family functioning, and self-efficacy level

In this study, the psychological resilience score of prostate cancer patients was (62.04 ± 15.47), the family functioning score was (6.77 ± 2.15), and the self-efficacy score was (25.24 ± 4.45). Age, place of residence, education, marital status, average monthly family income and treatment modality had significant differences in psychological resilience scores (*P* < 0.05). In contrast, only educational level, marital status, average monthly household income and treatment modality had significant differences in family functioning scores (*P* < 0.05). All factors except age had significant differences in self-efficacy scores (*P* < 0.05), as shown in Table 1.

### 3.3 Correlation analysis of psychological resilience, family functioning, and self-efficacy

Pearson correlation analyses for the main variables are presented in Table 2. These correlations provide initial support for the previously hypothesized relationships. These results indicate that psychological resilience was positively correlated with family functioning (*r* = 0.526, *P* < 0.01) and with self-efficacy (*r* = 0.378, *P* < 0.01), and similarly, family functioning and self-efficacy were positively correlated (*r* = 0.358, *P* < 0.01).

**Table 2:** Correlation analysis of psychological resilience, family function, and self-efficacy.

**Variable**	**Psychological resilience**	**Family functioning**	**Self-efficacy**	**mean**	**SD**
Psychological resilience	1	–	–	62.04	15.47
Family functioning	0.526^**^	1	–	6.77	2.15
Self-efficacy	0.378^**^	0.358^**^	1	25.24	4.45

^**^At a 0.01 level (double tails), the correlation was significant.

### 3.4 Mediation test

The aim of this study was to examine the relationship between self-efficacy, family functioning and psychological resilience in prostate cancer patients. The Process plug-in was used to analyze the mediating role of self-efficacy in the relationship between family functioning and psychological resilience in prostate cancer patients. A sample of 5,000 was bootstrapped with 95% confidence intervals (95% CI), with family functioning as the independent variable, self-efficacy as the mediator, and psychological resilience as the dependent variable.

**Figure 1:**
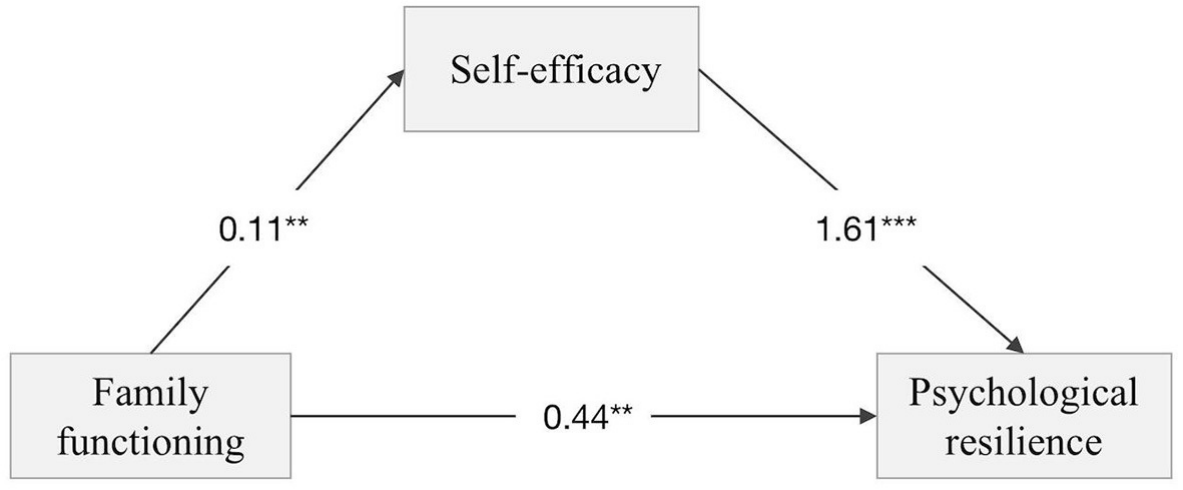
A mediation model of the role of self-efficacy in the relationship between Family functioning and Psychological resilience in patients with prostate cancer. ^**^Indicates that *P* < 0.01 and ^***^states that *P* < 0.001.

The results showed that family functioning positively predicted psychological resilience in prostate cancer patients (β = 0.43, *p* < 0.001). Navigating social support positively predicted self-efficacy (β = 0.11, *p* < 0.001). The confidence intervals for each pathway did not include 0, suggesting that self-efficacy partially mediates the relationship between family functioning and psychological resilience, revealing that the effect of family functioning on psychological resilience in prostate cancer patients is partially due to the influence of self-efficacy. The results show that the value of the mediation effect is 0.186, which accounts for 42.56% of the total effect, as shown in Table 3. The mediation model is presented in Figure 1.

**Table 3:** Bootstrap analysis of the mediating effect of self-efficacy between family function and psychological resilience.

**Project**	**Standardized effect value**	**SE**	**Bootstrap 95% CI**		**Relative effect value (%)**
			**Lower limit**	**Superior limit**	
Direct effect	0.251	0.030	0.021	0.127	57.44
Indirect effect	0.186	0.051	0.497	0.640	42.56
Total effect	0.437	0.050	0.562	0.774	

## 4 Discussion

This study confirmed Hypothesis 1: There is a positive relationship between family functioning, psychological resilience and self-efficacy in prostate cancer patients, and also confirmed Hypothesis 2: Self-efficacy can mediate the relationship between family functioning and psychological resilience in prostate cancer patients, which means that family functioning directly affects psychological resilience and can also affect it indirectly through self-efficacy.

This study found that the psychological resilience of prostate cancer patients is at a moderately low level compared to normal people and is lower than that of prostate cancer patients in Australia (Sharpley et al., 2014). Since most of the individuals in this study stated they are the main source of income for their family, their diagnosis of prostate cancer likely leads to self-isolation and increased stress (Gentili et al., 2022) as the Chinese cultural concept of masculinity is challenged (Oliffe, 2023). Compared to the general population, prostate cancer patients are firstly physiologically threatened by the cancer, and at the same time, the complications brought about by the treatment, such as body image disorders, pain and other triggers of psychological stress, reduce the level of mental health of patients. Studies have shown that patients with high psychological resilience have better self-motivation and independence, are less depressed (Sharpley et al., 2021), and also have better physical, emotional and social functioning and quality of life (Seiler and Jenewein, 2019), whereas patients with low resilience suffer more whereas patients with low resilience suffer greater distress (Macía et al., 2021) and cancer-related fatigue (Öcalan and Üzar-Özçetin, 2022), therefore attention to and understanding of psychological resilience can help patients manage the difficulties associated with cancer and motivate them to better participate in and adapt to treatment (Kim et al., 2019).

Modern stress theory suggests that a good external environment helps patients to resist setbacks and hardships and acts as a buffer (Ogilvie et al., 2015). Most of the patients in the study had good family functioning, which is positively associated with psychological resilience, suggesting that patients with better family functioning receive better family support. Family functioning may act as a protective factor, enabling patients to value and strengthen pre-existing social ties (Zhang Y. et al., 2023).

In our study, family functioning increased psychological resilience through self-efficacy, which is consistent with Karademas et al. (2023) findings that self-efficacy can act as a mediating variable to influence psychological resilience. From the couple's perspective, complications of prostate cancer such as erectile dysfunction and loss of sexual function may affect the couple's intimacy, leading to family disharmony (Gupta et al., 2023), and patients may perceive themselves as a burden to the family, thus lacking confidence in recovery. From the perspective of overall family support, in China, influenced by the traditional values of Confucius and Mencius, it is the responsibility and duty to care for a loved one as a family member, and the care and comfort of the family can help them recover as quickly as possible (Peng et al., 2023). Patients with good family functioning and high levels of intimacy can better draw energy from their families and promote better self-change and confidence in their ability to manage themselves (Gibbons et al., 2019).

However, as the disease progresses, focusing on family functioning alone may not be sufficient to help patients recover from the stress of the disease. In addition to family problems, patients may face psychological changes brought about by economic changes, job changes, social exclusion, etc. A high level of self-efficacy can be effective in reducing the stress that patients themselves experience, helping them to build psychological protection, cope positively with difficult situations, and manage anxiety well. A study of early-stage cancer patients found that higher levels of self-efficacy were associated with better levels of psychological resilience (Liu K. L. et al., 2022). High levels of self-efficacy make it easier for patients to assess their state in response to a crisis (Chien et al., 2022), pay more attention to the demands of stressful situations, feel more effective in the face of cancer-induced changes and challenges, and promote better psychological adjustment and high levels of resilience (Freire et al., 2020). Prostate cancer patients with high self-efficacy tend to have greater confidence in facing the disease, they believe that this cancer can be beaten, that a radical cure can be achieved in most people with surgery, and that with exercise their postoperative standard of living can be restored to normal pre-morbid status. It follows that prostate cancer patients with well-functioning families experience higher levels of self-efficacy and are able to use this to strengthen their own levels of psychological resilience.

European Association of Urology and the American Association of Urology have recognized a decline in the quality of life and mental health of prostate cancer patients, with their psychological state becoming an increasingly important factor in clinical assessment (Vyas et al., 2023). This study has several implications for clinical practice with prostate cancer patients. Firstly, the mediating role of self-efficacy between family functioning and psychological resilience suggests the need to focus on the patient's own and family status, alongside the idea of empowering the patient to be more assertive. In addition, self-efficacy can be used as a target for intervention to help patients make better use of their own and their family's strengths in coping with their illness. The current study confirms the importance of external support and personal protection for patients' mental health, and future interventions should focus on modifying negative psychological factors to help patients in their recovery process. The mental health level of prostate cancer patients is affected by a variety of internal and external factors, so it is suggested that the community should identify, screen and help patients with poorer health as early as possible, and that the government can set up a community health service program to incorporate psychological counseling into community health education. Healthcare professionals should also focus on patients' mental health and informal social support. Family members serve as important social support for patients, and often the companionship of spouses and the care of children are the spiritual pillars that support patients through the treatment phase. Therefore, during the treatment and rehabilitation phases, family participatory interventions can be borrowed and segmented care interventions based on timing theory can be implemented, with family members encouraging and supporting patients throughout the rehabilitation process, increasing closeness between family members and building positive family beliefs. Interventions that focus on emotional interaction, such as couple intimacy enhancement therapy (Reese et al., 2019) and binary coping (Terrill et al., 2023) have been shown to improve negative emotions and increase psychological resilience in both partners by improving communication. Self-efficacy theory suggests that self-efficacy is key in determining whether a person will adopt new healthy behaviors (Bandura, 1977). Self-confidence is a key factor in self-management for patients following cancer treatment. The diagnosis and treatment of cancer makes individuals vulnerable and reduces their self-confidence. Rebuilding self-confidence helps patients to better cope with problems caused by cancer and its treatment, thereby improving their outcomes (Foster and Fenlon, 2011). Active self-management interventions can not only improve patients' self-confidence in symptom management, but can also help to improve adaptation to the disease. Self-management interventions for prostate cancer patients led by professional caregivers have been demonstrated to improve psychological resilience by assisting patients with self-management through mobile self-management apps, distribution of health education brochures, and provision of professional health support (Chien et al., 2023). Additionally, Acceptance and Commitment Therapy has been shown to help avoid unpleasant personal experiences related to cancer, encourage the choice to accept these events, identify important personal values and self-efficacy, and promote a commitment to act on these values (Li et al., 2021), to help enhance psychological resilience and health outcomes for cancer patients.

## 5 Conclusion

From the perspective of positive psychology, which focuses on exploring and cultivating a variety of positive psychological resources to enhance an individual's positive emotions and competencies (Liu L. et al., 2022), this study examined the relationship between self-efficacy, family functioning, and psychological resilience as well as their effects on patients with prostate cancer. This study suggests that physicians should not only provide regular psychological counseling to prostate cancer patients, but also focus on assessing the patients' family situation and self-efficacy, and combine the interactions of the three, to clearly understand patients' motivations and outcomes, to broaden their understanding of the patients (Sebri et al., 2022), and to work with a multidisciplinary team, including psychologists, to implement more detailed mental health education and to enhance the patients' confidence in facing the treatment of their disease.

## 6 Study limitations

This study has several limitations. First, the study was cross-sectional and could not reflect causality compared to cohort studies; second, our study only examined the mediating role of self-efficacy, which may be influenced by more external factors as well as personal factors, as suggested by the RIM model, and the mediating role of variables such as self-esteem and hope could be considered at a later stage. Thirdly, the vast majority of the sample included in this study were treated surgically, with less endocrine treatment and active observation, and the population could be further expanded at a later stage.

## Data availability statement

The original contributions presented in the study are included in the article/supplementary material, further inquiries can be directed to the corresponding author.

## Ethics statement

The studies involving humans were approved by Yancheng Third People's Hospital. The studies were conducted in accordance with the local legislation and institutional requirements. The participants provided their written informed consent to participate in this study. Written informed consent was obtained from the individual (s) for the publication of any potentially identifiable images or data included in this article.

## Author contributions

YZ: Writing—original draft. HS: Project administration, Supervision, Writing—review & editing. CW: Investigation, Methodology, Writing—review & editing. HC: Data curation, Investigation, Writing—review & editing. YS: Investigation, Methodology, Writing—review & editing. WS: Data curation, Investigation, Methodology, Writing—review & editing. LW: Formal analysis, Investigation, Methodology, Writing—review & editing. QL: Conceptualization, Data curation, Project administration, Resources, Validation, Writing—review & editing.
